# Breathing new life into pain management: a systematic review of nebulised ketamine for analgesia

**DOI:** 10.1186/s13049-025-01501-4

**Published:** 2025-12-04

**Authors:** Daniel Kirk, Emily Whiles, Anita Jones, Christopher Edmunds

**Affiliations:** 1 Emergency Department, Peterborough City Hospital, North West Anglia Foundation Trust, Edith Cavell Campus, Bretton, PE3 9GZ Peterborough, UK; 2 University of Warwick, Warwick, UK; 3 East Anglian Air Ambulance, Gambling Close, Norwich Airport, Norwich , NR6 6EG UK; 4 University of East Anglia, Norwich , UK; 5 Dudley Group Foundation Trust, DY1 2HQ, Dudley, UK

**Keywords:** Nebulised ketamine pain management analgesia pain

## Abstract

**Background:**

Acute pain accounts for 60–90% of presentations to the emergency department (ED), with 20–40% of patients reporting severe pain. Current management practices, including simple analgesics, opiates and anti-inflammatory drugs, are often inadequate or slow to reach peak effect, necessitating the exploration of alternative analgesics. Ketamine, acting primarily through N-methyl-D-aspartate (NMDA) receptor antagonism, presents a promising alternative due to its rapid onset. However, its nebulised form remains underutilised in clinical practice.

**Aims and objectives:**

This review evaluates the efficacy of nebulised ketamine in reducing pain in adult ED patients, alongside its side effect profile, optimal dosing, and potential as an alternative or adjunctive analgesic compared to other treatments.

**Methods and design:**

A systematic review utilising the PRISMA guidelines was conducted. Searches were carried out in Medline, Embase, PubMed, Science Direct, google scholar and Ovid databases from 2010 to May 2024, including studies containing objective analysis of pain control with nebulised ketamine. A two-sample t-test was used to assess statistical significance. Quality assessment was performed using the CASP tool, and bias was evaluated using the ROBINS-I and ROB2 tool.

**Results:**

Of 99 articles, 9 (5 randomised controlled trials, 3 case series and 1 case report) totalling 453 patients were included. All studies suggested improvement in pain scores with nebulised ketamine, with an average reduction of 42.5% and 70.4% over a 15 and 120-minute period respectively (*p* < 0.0001). Higher doses (1 mg/kg, 1.5 mg/kg) did not significantly improve pain compared to lower doses (0.7 mg/kg 0.75 mg/kg), with similar overall reductions reported across all four dosing regimens (*p* < 0.0003 or 0.0001). Nebulised ketamine was non-inferior to intravenous (IV) morphine, IV ketamine, nebulised dexmedetomidine, and Entonox, and had fewer side effects.

**Conclusion:**

Nebulised ketamine offers a viable alternative for pain management in emergency settings, providing effective analgesia with a favourable safety profile. Further multicentre trials with larger populations are recommended to confirm these findings and establish standardised dosing protocols for consideration in national guidance.

## Introduction

Acute pain is one of the most common presenting complaints to the Emergency Department (ED) accounting for 60–90% of ED attendances [1], with severe pain reported in 20–40% of cases [2]. Consequently, analgesia is a crucial component of emergency medicine and fundamental to patient management. Recent studies highlight deficiencies in current pain management practices within the ED, emphasising the urgent need for safer, faster-acting and more reliable analgesic options [3].

Adequate analgesia not only provides significant physiological and psychological relief to patients but also facilitates more accurate clinical assessments [4, 5], enables the performance of necessary but painful procedures, and ultimately improves patient outcomes [6]. Pain levels are commonly assessed using validated tools such as the verbal Numeric Rating Score (NRS) and the Visual Analogue Scale (VAS), both of which utilise an 11-point scale ranging from 0 (no pain) to 10 (severe pain), with patients selecting a number for the NRS, and placing a mark on the 10 cm line for the VAS.

**Fig. 1:**
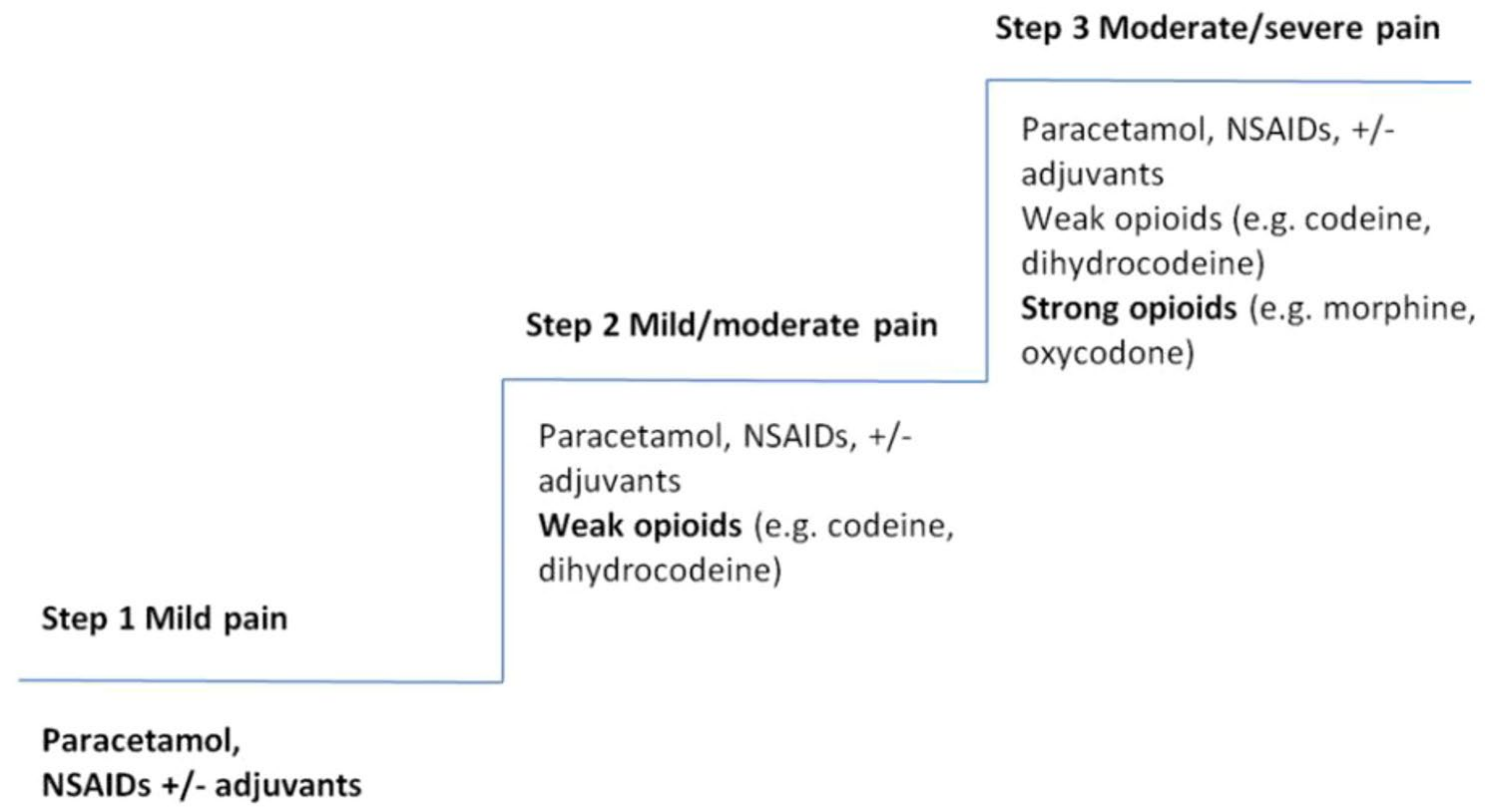
[7] WHO Pain ladder guidance for administering appropriate analgesia according to pain severity

The World Health Organisation (WHO) pain ladder [7] (Fig. 1) serves as a guide for administering appropriate analgesia according to pain severity. Regular reassessment of pain scores, ideally every 15–30 min, is imperative to review adequacy of analgesia and identify the need for further intervention, including non-pharmacological approaches [8].

Presently, opioid analgesics are used in the ED for managing moderate to severe, acute or chronic pain. However, growing awareness and frequent reporting of their adverse effects on patients, ranging from common side effects like nausea to more severe issues such as respiratory depression, has led to frequent underdosing, and therefore reduced efficacy [9, 10]. This has prompted a growing emphasis on identifying safer, more effective alternatives. Several of these have been examined in both research and clinical literature. Notably, the European Society for Emergency Medicine (EUSEM) reviewed the use of analgesics in the ED and issued recommendations advocating for a multimodal, stepwise approach to pain management, prioritising early pain assessment, non-opioid options when appropriate, and individualised treatment plans [11]. Sin et al. similarly concluded that non-opioids can serve as safe and effective alternatives for managing acute pain in the ED [12]. Lindbeck et al. [13] provided additional guidelines for pre-hospital care, emphasising the broad range of effective analgesic options available beyond opioids.

Entonox (a 50/50 mix of nitrous oxide and oxygen), is an inhaled analgesic used in the ED [31]. Although its analgesic mechanisms are not fully understood, they are thought to involve opioid-like effects at the spinal cord neuromodulators, as well as anxiolytic and anaesthetic effects similar to benzodiazepines, targeting GABAA and NMDA receptors [4, 14]. Nebulised dexmedetomidine is a selective alpha-2 adrenoceptor agonist, offering pain relief by inhibiting noradrenaline release and interrupting pain signal transmission [15]. Karlow et al. [16], describes Ketamine as a non-competitive NMDA/glutamate receptor antagonist and is another promising alternative. It reduces pain by mitigating central sensitisation, hyperalgesia and the “wind-up” phenomenon of peripheral C fibres, particularly at the spinal cord and central nervous system [17]. Although ketamine has traditionally been administered intravenously, nebulised ketamine is emerging as an alternative when intravenous (IV) access is challenging [18]. Dove et al. [19] conducted a Randomised Controlled Trial (RCT) comparing three doses of nebulised ketamine, demonstrating that *they all* effectively relieved pain without significant side effects.

Multiple publications have investigated the role of nebulised ketamine in various settings, including pre-hospital care [20], paediatric pain management [21], postoperative sore-throat management [22], and ED analgesia [23]. Despite its widespread use globally, nebulised ketamine is not yet licensed within the National Health service (NHS), National Institute of Health and Care Excellence (NICE) guidelines, or the British National Formulary (BNF). While several RCT’s, studies and reports have been published, there is currently no systematic review for reference. This study aims to analyse and critically review the literature to evaluate the efficacy and safety profile of nebulised ketamine, assessing its potential as an alternative or adjunctive analgesic in the ED.

## Methods

The protocol was registered in the international prospective register of systematic reviews (PROSPERO) on 20/06/2024, registration number 560320. The Preferred Reporting Items for Systematic Reviews and Meta-Analyses (PRISMA) guidelines [24] were adhered to throughout this study.

### Eligibility criteria

#### Inclusion criteria

Publications that review the use of nebulised ketamine within the ED or pre-hospital environment for acute or chronic pain; patients aged 18 years or more; measurement of pain using a quantitative standardized tool before and after intervention.

#### Exclusion criteria

Publications published before 2010; not published or translated into English; conference proceedings, letters or meeting abstracts; incomplete data (measurements not documented for either before, after the intervention or both).

### Information sources and search strategy

A literature search of the following six databases were conducted: Medline, Embase, PubMed, Science Direct, Google Scholar and Ovid databases from January 2010 to May 2024. A combination of the following keywords and Medical Subject Headings (MeSH) terms were used in the search strategy: “ketamine,” “nebulised,” “nebulized”, “analgesia,” “pain,” “Emergency department,” “pain management”.

### Study selection

Publications identified by the search strategy were assessed for inclusion; duplicates were removed and the remaining studies and reports were screened using their titles and abstracts. Any studies not fitting the eligibility criteria were excluded. Full text articles were obtained of the residual articles and the final publications were selected.

### Data collection and analysis

The data from each of the included studies were extracted into a standardised format specific to the primary and secondary outcomes, comprising of design, demographics, interventions, outcomes, measures of effect and the pain scores pre-administration of treatment, and at all subsequent recorded times. The results were analysed in subgroups regarding the analgesic administered. Patients receiving nebulised ketamine were further divided according to dose administered. The results from the 11 point NRS and VAS were accumulated as both recorded the same 0–10 pain scores. Outcomes were reported as means, percentage reduction in pain scores from baseline and narratively for side effects. They were further analysed using a two-sample t-test. Standard deviation was used for a population, and equal variance was assume for all standard deviations which were < 2. A p-value of < 0.05 was considered statistically significant. For Outcome 1 and 2.1, pain scores at recorded times were compared to the baseline pain score at 0 min. For Outcome 2.2 the pain scores at the given time were compared between nebulised ketamine and the comparative medication. The European Medicines Agency definition of adverse reactions and frequency of undesirable effects were used for all medications [25].

Throughout all studies there was no standardisation of inhalation time for the nebulisation or recording of actual treatment time.

### Outcomes

#### Primary outcome

- To assess the efficacy of nebulised ketamine in reducing pain.

#### Secondary outcomes

- To analyse the effective dose of nebulised ketamine.
- To compare the analgesic efficacy of nebulised ketamine with IV morphine, IV ketamine, Entonox and Dexmedetomidine for moderate to severe pain.
- To assess and compare reported side effects amongst the different treatments.

### Quality assessment

A quality assessment of included studies was carried out using the Critical Appraisal Skills Programme (CASP) checklist [26]. Risk of bias was calculated using version 2 of the Cochrane Risk of Bias Tool (ROB2) [27] for the randomised trials, and the Risk of Bias in Non-randomised Studies- of Interventions tool (ROBINS-I) [26] for the case series and case study.

## Results

### Study selection

**Fig. 2:**
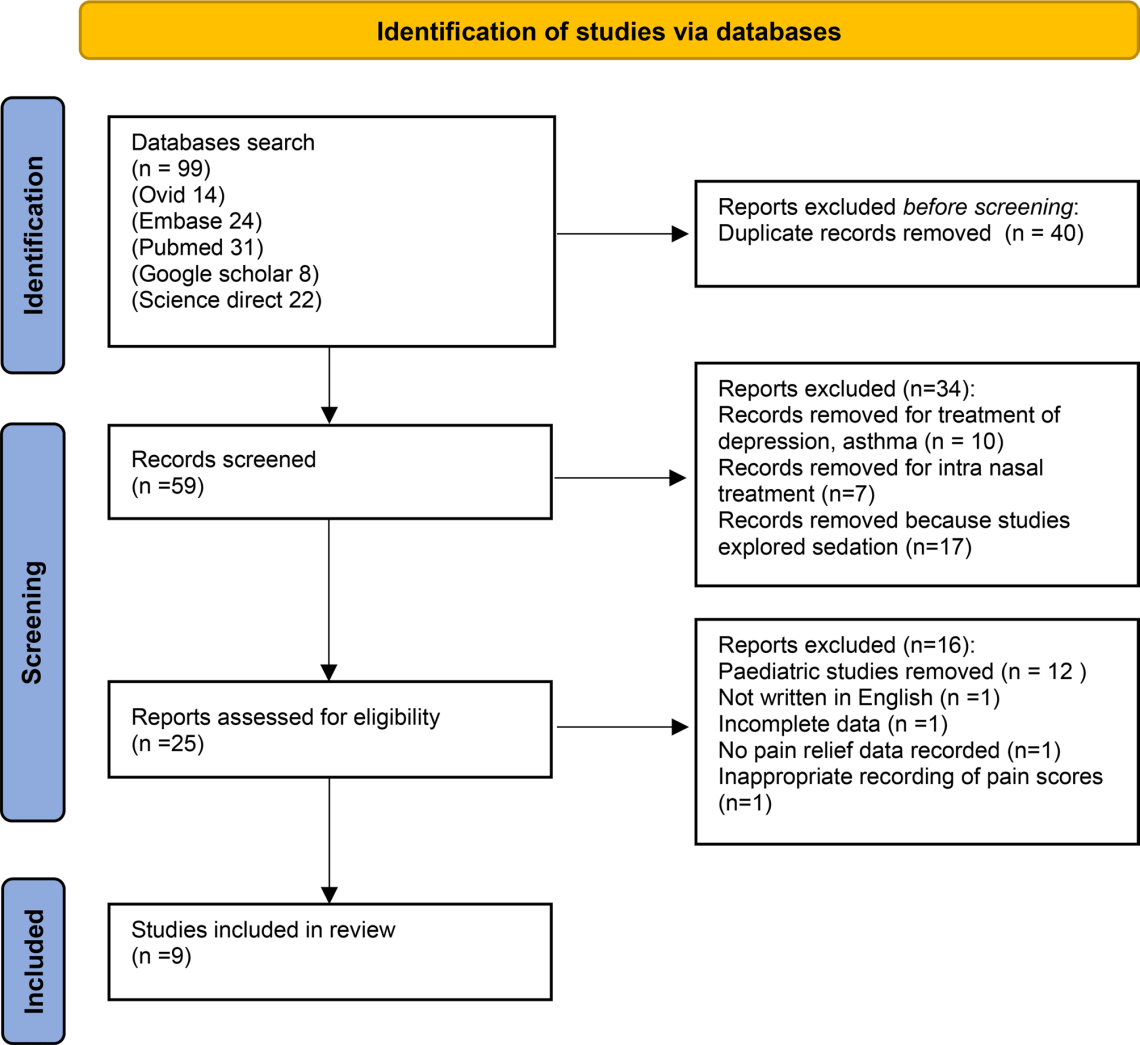
PRISMA flowchart illustrating the included and excluded studies

A systematic search of the databases revealed 99 articles. 40 duplicates were removed leaving 59 articles to be screened. Of these, 17 were removed at title screening due to non-fulfilment of the inclusion criteria, and a further 17 were removed following abstract assessment. The remaining 25 articles were reviewed using the full text and a further 16 studies were excluded leaving nine articles to be used in this review (Fig. 2).

### Study characteristics

An extended table of the study characteristics can be found in Appendix 8.

#### Study design

The nine studies included in the review consisted of five double blind RCT’s, three case series and one case study all published between 2020 and 2024. Two studies took place pre-hospital and seven were in the ED. They were all single centered, spanning the United States of America, Malaysia, Thailand and Iran.

#### Study population

Data were gathered from 453 patients, 208 male and 245 female, between the ages of 18 and 79. They presented with a wide variety of pain including acute pain (traumatic and atraumatic): abdominal, flank, genitourinary, back, musculoskeletal, shoulder dislocation, fractures, headaches, sickle cell crisis and chronic pain: abdominal, musculoskeletal, back, and neuropathic.

#### Study interventions

In total, 296 patients were treated with nebulised ketamine. These were compared to 75 patients managed with IV ketamine, 46 with IV morphine, 23 with dexmedetomidine and 13 with Entonox. All patients received a treatment no patients received a placebo-control. Of the patients who were given nebulised ketamine, 13 received 0.7 mg/kg, 164 received 0.75 mg/kg, 71 received 1 mg/kg and 45 received 1.5 mg/kg. In all studies the injectable formulation of ketamine used was 50 mg/mL concentration made up to 5mls with normal saline and given with 5 L of oxygen.

#### Study outcomes

Seven studies used an NRS pain scoring system and two used VAS. Side effects of the medication received were noted and measured using the Richmond agitation-sedation scale (RASS) (Appendix 9) and the side effects rating scale of dissociative anaesthetics (SERSDA) (Appendix 10). The results from all studies were used for nebulised ketamine (*n* = 9) [19, 20, 23, 28–35], and data from Kampan et al [30], Nguyen et al. [23], Motamed et al. [33] and Arumugam et al. [32] were used for IV morphine, IV ketamine, dexmedetomidine and Entonox respectively.

### Bias

**Table 1:**
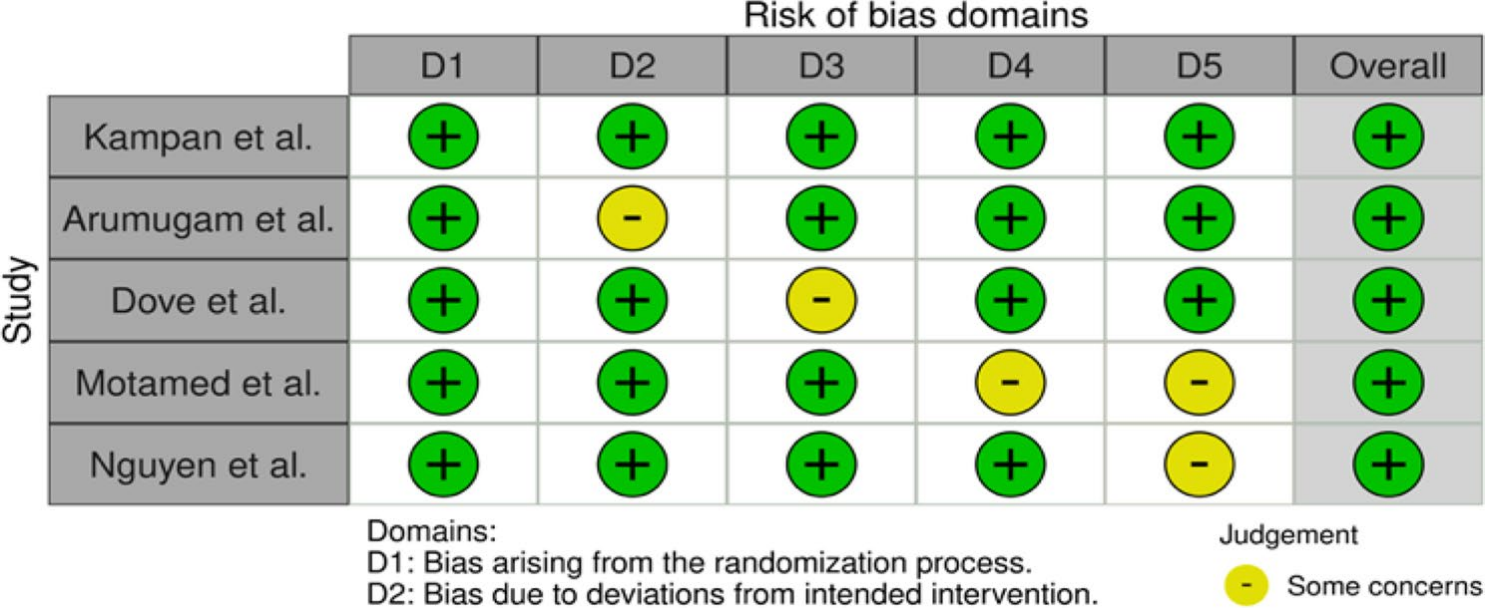
Risk of bias of the RCTs using the ROB2 tool

Summary tables assessing the risk of bias were constructed (Tables 1 and 2), demonstrating low risk of bias in the RCT’s, with some moderate or serious concerns in the case report and series.

**Table 2:**
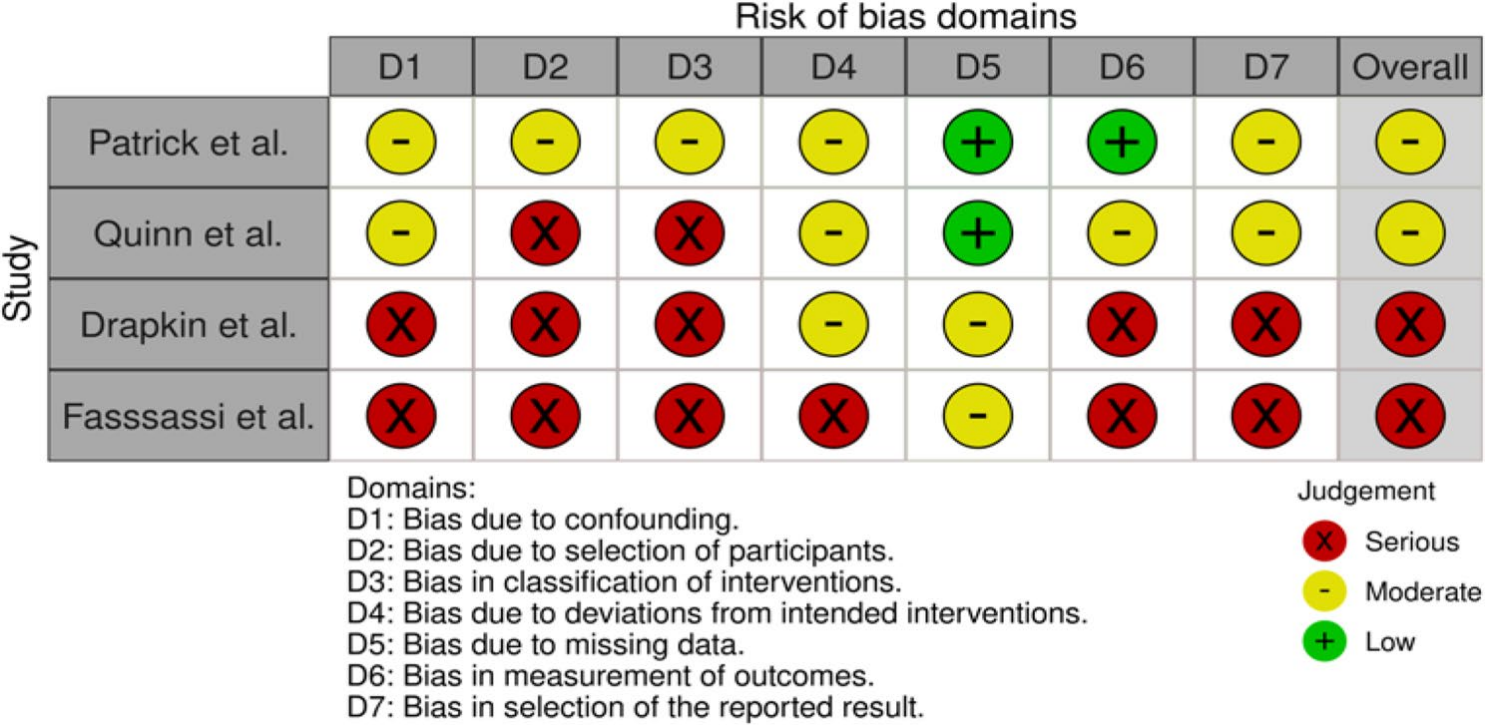
Risk of bias of the case report and case series using the ROBINS-I tool

### Effects of interventions

#### Primary outcome

##### To assess the efficacy of nebulised ketamine in reducing pain

Graph 3 and 4 demonstrate the mean reported pain scores and the mean percentage reduction respectively from the initial reported score prior to the patients receiving nebulised ketamine until 120 min had passed (*n*=296). There was an initial 42.5% reduction in pain at 15 min with an ultimate 70.4% reduction 120 min post administration. Table 3 illustrates that pain scores post administration of nebulised ketamine showed a clear and consistent, statistically significant reduction over time (*p*<0.0001).

**Fig. 3:**
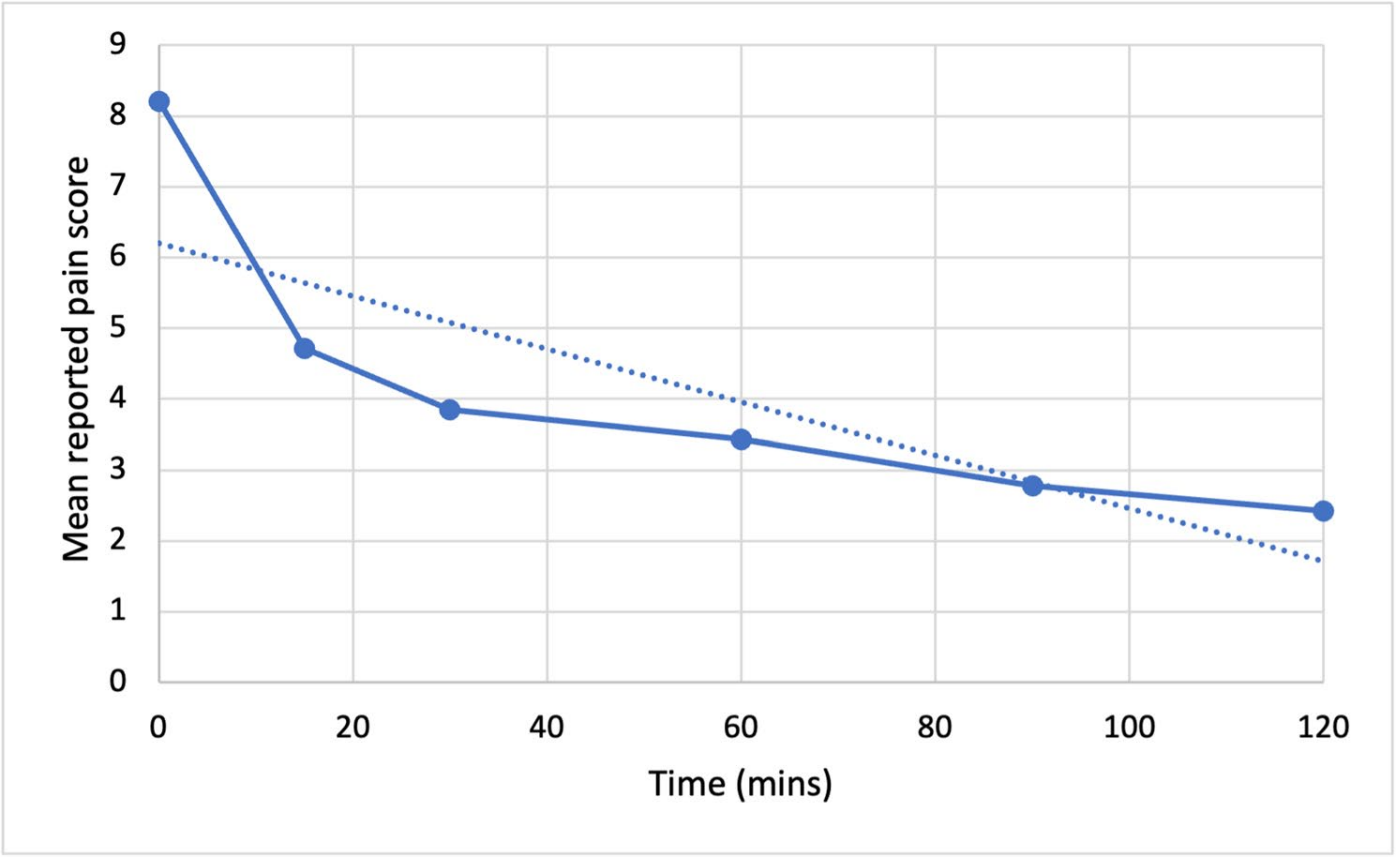
The mean reported pain scores over time with a trendline for reference

**Fig. 4:**
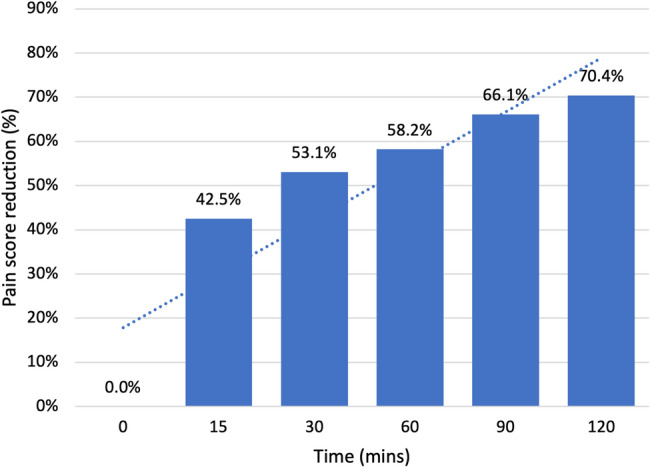
The mean percentage reduction in NRS pain scores over time with a trendline for reference

**Table 3:** Two-sample t-test of mean pain score reduction over time post nebulised ketamine administration

Time (min)	Mean pain scores	Standard deviation	t-value	95% Confidence Interval	*p*-value
0	8.21	1.08			
15	4.72	0.86	43.49	3.33+/-3.64	< 0.0001
30	3.85	0.82	55.31	4.20+/-4.51	< 0.0001
60	3.43	1.85	38.9	4.53+/-5.02	< 0.0001
90	2.78	1.23	57.07	5.24+/-5.61	< 0.0001
120	2.43	1.17	62.45	5.59+/-5.96	< 0.0001

#### Secondary outcomes

##### To determine the effective dose of nebulised ketamine

Graph 3 demonstrates the mean reported pain scores from the initial reported score prior to the patients receiving nebulised ketamine and at sequential time points post intervention.

Over the initial 30-minute period, patients who received 0.7 mg/kg (*n* = 13) experienced a mean pain score reduction of 43.8%. Those receiving 0.75 mg/kg (*n* = 164) had a 45.75% reduction, 1 mg/kg (*n* = 71) showed a 56.8% reduction, and 1.5 mg/kg (*n* = 45) demonstrated a 58% reduction. All dose groups had comparable baseline pain scores, and pain scores continued to be recorded up to 120 min post-administration. At 120 min, patients receiving 0.75 mg/kg (*n* = 164) showed a total pain score reduction of 68.75%, those receiving 1 mg/kg (*n* = 71) had a 61.3% reduction, and those receiving 1.5 mg/kg (*n* = 45) had a 66.6% reduction. Table 4 demonstrates that all doses significantly reduce pain scores at all points in time (*p*≤0.0003), with the lowest confidence intervals at higher doses. The 0.7 mg/kg, although significant, has the lowest overall t-values and the lowest p-value than the other measured doses.

**Fig. 5:**
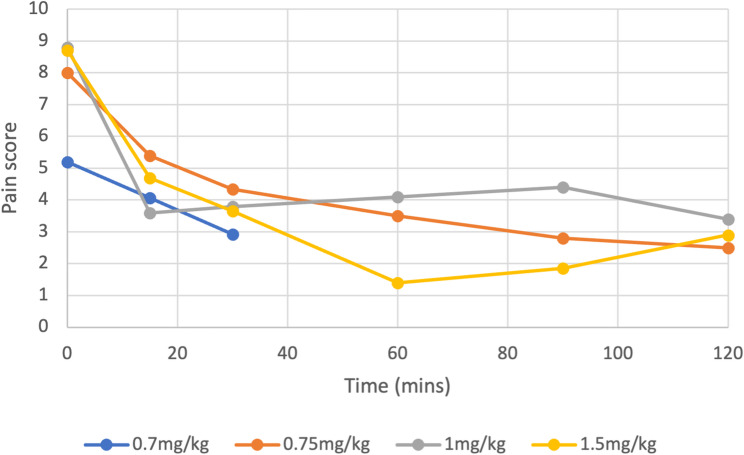
The mean pain scores reported for the four doses of nebulised ketamine administered over time

**Table 4:** Two-sample t-test of mean pain score reduction over time post nebulised ketamine administration for 0.7 mg/kg, 0.75 mg/kg, 1 mg/kg and 1.5 mg/kg

Dose (mg/kg)	Time (mins)	Mean pain score	SD	T-value	95% Confidence Interval	*P*-value
0.7	0	5.07	0.438			
	15	4.07	1.37	4.26	0.87+/-2.52	0.0003
	30	2.92	1.26	7.73	2.08+/-3.61	< 0.0001
0.75	0	8	0.44			
	15	5.4	0.93	32.3	2.44+/-2.75	< 0.0001
	30	4.34	0.82	50.9	3.55+/-3.84	< 0.0001
	60	3.5	1.81	30.9	4.28+/-4.78	< 0.0001
	90	2.8	1.15	54	5.01+/-5.38	< 0.0001
	120	2.6	1.07	59.7	5.22+/-5.57	< 0.0001
1	0	8.8	0.56			
	15	4.4	0.99	32.5	4.13+/-4.66	< 0.0001
	30	3.5	0.66	51.59	5.09+/-5.50	< 0.0001
	60	5.6	2.92	9.06	2.50+/-3.89	< 0.0001
	90	2.2	2.20	24.4	6.06+/-7.13	< 0.0001
	120	1.7	1.70	33.42	6.68+/-7.52	< 0.0001
1.5	0	8.8	0.14			
	15	5.2	0.85	28	3.34+/-3.85	< 0.0001
	30	4	0.65	48.4	4.60+/-4.99	< 0.0001
	60	1.9	1.64	28.1	6.41+/-7.38	< 0.0001
	90	2.4	1.35	31.6	5.99+/-6.80	< 0.0001
	120	2.2	1.45	37.3	7.66+/-8.53	< 0.0001

##### Compare the analgesic efficacy of nebulised ketamine with other commonly used medications for moderate to severe pain

Graph 6 demonstrates pain reductions for IV morphine (*n* = 46), IV ketamine (*n* = 75), dexmedetomidine (*n* = 23) and Entonox (*n* = 13) compared to nebulised ketamine over time. This is further broken down into graphs 7–7, and t-values assessed in Tables 5, 6, 7 and 8. At 30 min, the patients receiving Entonox had a pain score reduction of 53.3% compared to 26.9% reduction IV morphine, 21% nebulised dexmedetomidine, 56% IV ketamine and 53.1% reduction for nebulised ketamine. At 60 min, the patients receiving IV morphine had a pain score reduction of 41% compared to 49.4% reduction nebulised dexmedetomidine, 59.7% IV ketamine and 58.2% reduction for nebulised ketamine. At 120 min, the patients receiving IV morphine had a pain score reduction of 57.6% compared to 59.7% IV ketamine and 70.4% reduction for nebulised ketamine. The tables and t-tests demonstrate that nebulised ketamine, IV ketamine and IV morphine had similar pain scores prior to treatment administration (*p* < 0.948 and 0.244 respectively), allowing further review of pain scores over time. IV ketamine was faster acting than nebulised ketamine at 15 min (*p* < 0.0001), with no significant difference in pain scores between 30 and 60 min (*p* < 0.24;*p* < 0.63) and nebulised ketamine producing significantly lower pain scores at 90 and 120 min (*p* < 0.0001; *p* < 0.0001). Nebulised ketamine produced lower pain scores at all times in the IV morphine comparison (*p* < 0.001). For dexmedetomidine and entonox, the initial pain scores were significantly different to the patients managed with nebulised ketamine (*p* < 0.0001), prohibiting further analysis.

**Fig. 6:**
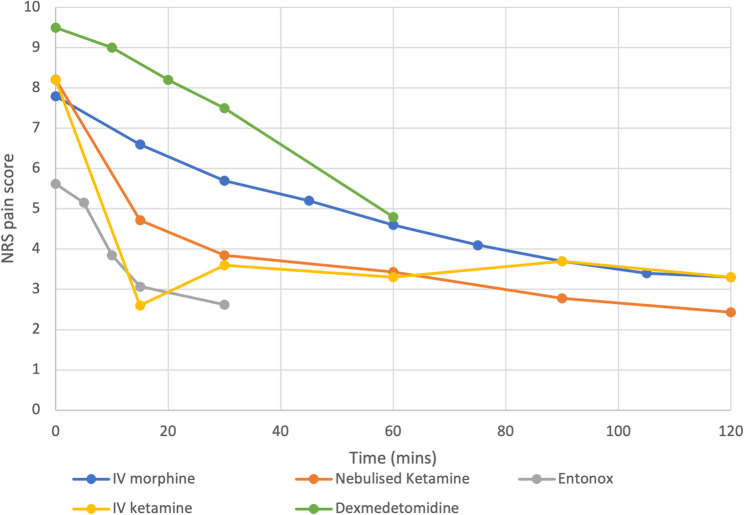
The mean pain scores reported for comparative analgesics over time

**Fig. 7:**
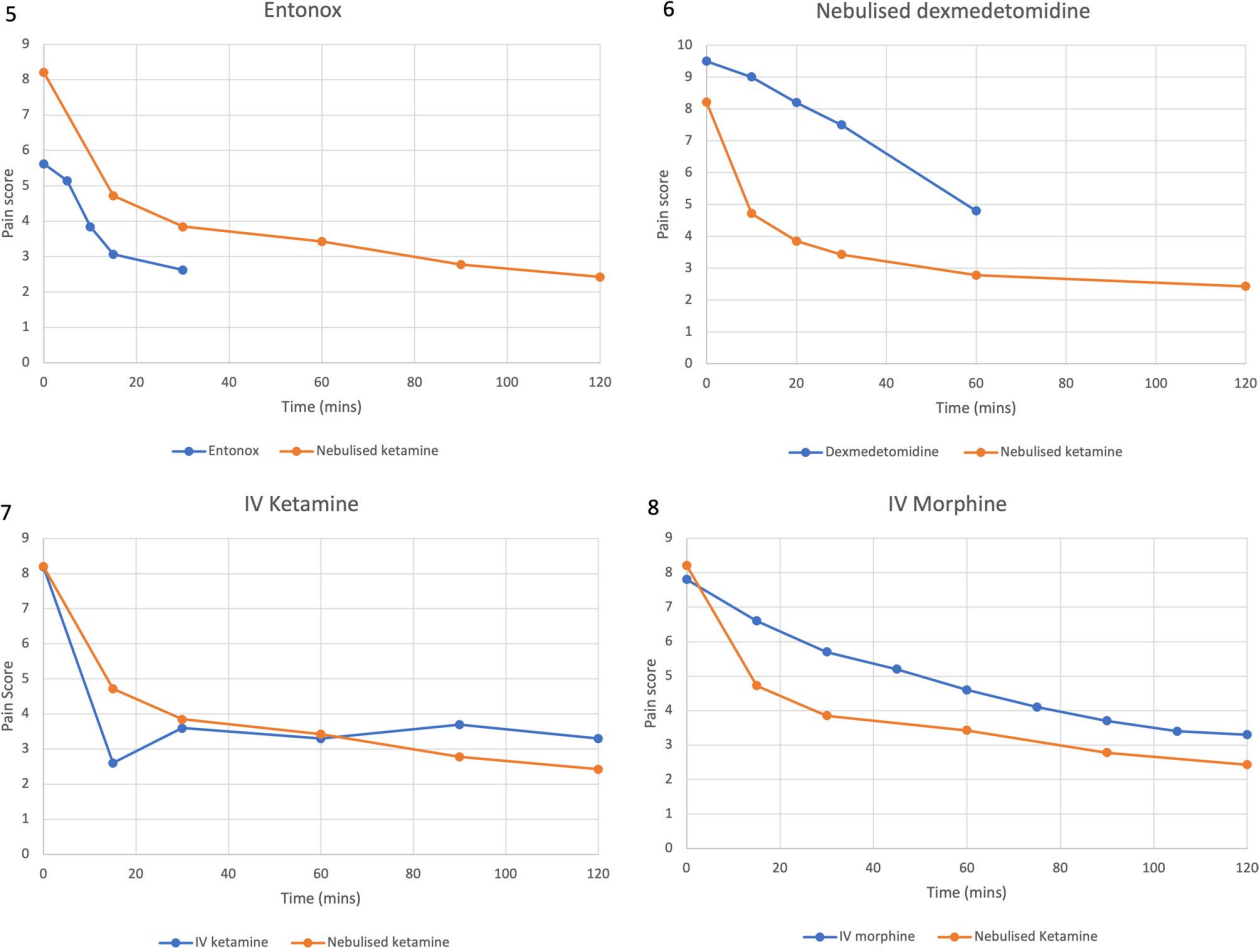
Demonstrating the mean pain scores reported for nebulised ketamine compared to Entonox (top left), nebulised dexmedetomidine (top right), IV ketamine (bottom left) and IV morphine (bottom right) over time

**Table 5:** The difference between mean pain scores reported for nebulised ketamine and entonox over time

Time (min)	Nebulised ketamine mean pain score	Entonox mean pain score	Standard deviation nebulised ketamine	Standard deviation Entonox	t-value	95% confidence interval	*p*-value
0	8.21	5.62	1.08	0.506	7.82	1.90+/-3.27	< 0.0001
15	4.72	3.07	0.86	0.759	5.18	0.99+/-2.3	< 0.0001
30	3.85	2.62	0.82	0.63	4.28	0.63+/-1.82	0.0003

**Table 6:** The difference between mean pain scores reported for nebulised ketamine and Dexmedetomidine over time

Time (min)	Nebulised ketamine mean pain score	Dexmedetomidine mean pain score	Standard deviation nebulised ketamine	Standard deviation dexmedetomidine	t-value	95% confidence interval	*p*-value
0	8.21	9.5	1.08	0.96	5.55	1.7+/- -0.83	< 0.0001
30	3.85	7.5	0.82	1.44	19.2	4.02 +/-3.27	< 0.0001
60	3.43	4.8	1.85	1.68	3.44	2.15+/-0.58	0.0007

**Table 7:** The difference between mean pain scores reported for nebulised ketamine and IV ketamine over timetable 6: the difference between mean pain scores reported for nebulised ketamine and Dexmedetomidine over time

Time (min)	Nebulised ketamine mean pain score	IV ketamine mean pain score	Standard deviation nebulised ketamine	Standard deviation IV ketamine	t-value	95% confidence interval	*p*-value
0	8.21	8.2	1.08	1.6	0.06	0.29+/-0.31	0.948
15	4.72	2.6	0.86	3.1	10.3	1.76+/-2.53	< 0.0001
30	3.85	3.6	0.82	3.3	1.17	-0.16+/-0.66	0.2418
60	3.43	3.3	1.85	2.8	0.48	-0.39+/-0.64	0.628
90	2.78	3.7	1.23	3.2	3.93	-1.3+/-0.46	< 0.0001
120	2.43	3.3	1.17	3	3.95	1-0.3+/-0.43	< 0.0001

**Table 8:** The difference between mean pain scores reported for nebulised ketamine and IV morphine over time

Time (min)	Nebulised ketamine mean pain score	IV morphine mean pain score	Standard deviation nebulised ketamine	Standard deviation IV morphine	t-value	95% confidence interval	*p*-value
0	8.21	7.8	1.08	1.5	2.26	0.05+/-0.76	0.244
15	4.72	6.6	0.86	2.2	10.4	-2.23+/- -1.52	< 0.0001
30	3.85	5.7	0.82	2.3	10.3	-2.20+/- -1.4	< 0.0001
60	3.43	4.6	1.85	2.3	3.8	-1.76+/- -0.57	< 0.0001
90	2.78	3.7	1.23	2.3	4.09	-1.36+/--0.47	< 0.0001
120	2.43	3.3	1.17	2	4.18	-1.27+/-0.46	< 0.0001

##### To assess and compare reported side effects among the different treatments

Of the 296 patients receiving nebulised ketamine 12.7% (*n* = 36) reported side effects, with the majority showing RASS scores of ±1 and SERSDA scores of 2. The most frequently reported side effects were dizziness and fatigue, affecting 97.2% (*n* = 35) of the affected participants, and 2.8% (*n* = 1) reported a mood change, characterised by feelings of relaxation and happiness. No serious adverse reactions were recorded,. The occurrence of these side effects were consistent across all dose regimens, with higher doses reporting the same incidence of adverse features as lower doses. Across all studies, patient vital signs were monitored during the administration of nebulised ketamine, and no significant changes were observed.

Out of the patients receiving Entonox, 53.85% (*n* = 7) experienced adverse effects, primarily dizziness. In the morphine group, 19.5% (*n* = 9) reported dizziness, 17.3% (*n* = 8) experienced nausea, and 4.34% (*n* = 2) reported general discomfort. No data were collected on side effects of the patients receiving dexmedetomidine. IV ketamine reported higher incidence of side effects when compared to nebulised ketamine, including sedation, restlessness, dizziness and feelings of unreality up to 30 min post drug administration, with RASS scores of + 2 and SERSDA scores of 3 reported. More severe side effects, such as sedation, restlessness, dizziness, and feelings of unreality, were reported 30 min post-administration with intravenous ketamine than with nebulised ketamine.

#### Additional analyses

Across the included data for nebulised ketamine, 12.5% (*n* = 37) of patients required supplementary analgesia, most commonly morphine, fentanyl, tramadol, or ketorolac. However, inconsistent reporting of the timing, dosage and adherence to study protocols for these additional medications limits the interpretation of these findings.

A comparison between prehospital and (ED) administration also yielded inconclusive results. Of the 296 patients who received nebulised ketamine, only eight were treated prehospitally, demonstrating a mean pain reduction of 59.9% compared to 65.9% in the ED group. Due to the small prehospital sample, further analysis was not possible.

None of the included studies differentiated between acute and chronic presentations, preventing further evaluation of potential variations in response according to pain type.

## Discussion

This systematic review analysed data from nine studies evaluating the efficacy and safety of nebulised ketamine as an analgesic. The results suggest that nebulised ketamine significantly reduces pain scores while causing fewer side effects when compared to commonly used alternatives.

The primary outcome to analyse pain reduction post administration of nebulised ketamine revealed a consistent, statistically significant decrease in pain scores over time. Fassassi et al. [28], and Drapkin et al. [29], reported the most substantial pain reductions, through both studies were small case series with high risk bias. When interpreted with the remaining more methodologically robust data, the pooled findings support strong overall analgesic efficacy for nebulised ketamine.

Analysis of the dose–response relationship for nebulised ketamine demonstrated that all tested doses (0.7-1.5 mg/kg) achieved significant analgesia. The 0.7 mg/kg cohort showed a slower onset and less pronounced analgesic effect compared with higher doses, particularly 1.5 mg/kg achieved faster and more consistent pain reductions. These differ from Dove et al. [19], who reported no significant difference in pain relief between 0.75 mg/kg, 1 mg/kg, and 1.5 mg/kg. The variation may reflect methodological differences and smaller sample size. Although a lack of standardisation in nebulisation duration limits precisions overall, all regimens produced significant analgesia, indicating that despite minor differences in onset and magnitude, each dose of nebulised ketamine is capable of achieving clinically significant pain relief.

A comparative analysis was conducted as part of the review to evaluate the efficacy of alternative analgesics against nebulised ketamine. At 30 min IV ketamine, nebulised ketamine and Entonox achieved greater reductions in pain scores compared to IV morphine and nebulised dexmedetomidine. By 120 min nebulised ketamine, demonstrated superior overall percentage reduction in analgesia surpassing both IV ketamine and morphine. Pain scores between IV ketamine and nebulised ketamine were comparable at 30 and 60 min, indicating comparable efficacy during early stages. It produced significantly lower pains scores than IV morphine at all time points. Unfortunately, data for Entonox were only available up to 30 min and dexmedetomidine stopped at 60 min, limiting direct comparisons and long-term efficacy analysis of percentage pain reduction.

Kampan et al. [29], found that nebulised ketamine provided non-inferior analgesia with fewer adverse effects compared to IV morphine in elderly patients. Conversely, Azizkhani et al. [34], reported that IV morphine alone had a greater pain reduction at 15–30 min compared to nebulised ketamine combined with morphine. Arumugam and Mohammod [32] observed no significant difference in pain reduction between nebulised ketamine and Entonox. Nguyen et al. [23], compared nebulised and IV ketamine, finding both routes to be equally effective in reducing pain scores. Motamed et al. [33], compared nebulised dexmedetomidine with nebulised ketamine finding that whilst both treatments significantly reduced pain scores, dexmedetomidine had a faster action onset time, and therefore faster reduction of pain. Differences in outcomes between these RCTs and our analysis may stem from the broader data amalgamation and larger patient population in our review. However, our analysis was limited to only consolidating data for the nebulised ketamine arm due to the scarcity of research directly comparing other analgesics. Some RCTs showed potential for selection and blinding bias, such as those using convenience sampling or visibly different delivery devices. Overall, our results demonstrate that nebulised ketamine is at least non-inferior to the alternative analgesics reviewed and in some cases, superior in significantly reducing pain scores.

An analysis of adverse effects revealed a favourable safety profile. Only 12.7% of patients receiving nebulised ketamine reported side effects, which were consistently minimal. Importantly, no serious adverse reactions were recorded in any of the studies. Jonkman et al. [35] and Muhamad et al. [36] supported these findings in an observational study, concluding that nebulised ketamine was well tolerated with no serious side effects, even at higher doses and when considering psychomimetic or emergence phenomena. When compared with IV morphine, IV ketamine, and Entonox, nebulised ketamine was associated with fewer and less severe reactions. Although incomplete reporting in some studies limited direct comparison, overall there is good tolerability and low risk of significant adverse effects.

### Additional considerations

Information regarding the pharmacokinetics and bioavailability of nebulised ketamine is limited. Jonkman et al. [35], reported that nebulised ketamine has a longer half-life than IV ketamine and morphine, which may result in extended pain relief. They observed a time to reach maximum concentration of 15 to 22 min [36], aligning with our findings. However, the applicability of these results to the general population remains uncertain due to these studies utilising a small number of healthy volunteers, Drenth-van et al. [37] noted that drug concentration and bioavailability may be delayed in older individuals or those with comorbidities.

Yanagihara et al. [38], estimated the bioavailability of nebulised ketamine to be between 20 and 40%. However, all studies in our review utilised a Breath Actuated Nebuliser (BAN), which only reports a 4% loss, thereby increasing the drugs bioavailability and efficacy. This increased efficiency, coupled with the BAN’s precise and adjustable dosing, contributes to greater safety for both patients and healthcare providers [39, 40].

### Clinical implications and future research

The findings of this review indicate that nebulised ketamine could be a valuable addition to standard pain management protocols in the ED setting, offering clinicians and patients a non-invasive, effective, and safe alternative to traditional analgesics. Its use may be particularly beneficial for patients who are at risk for complications from opioids or other analgesics, as well as in situations where rapid, non-invasive pain relief is required.

However, the review also highlights the need for further research to confirm the findings and address the limitations of the current evidence base. Most of the studies included in the review were single-centered with relatively small sample sizes, which limits the generalisability of the results. A large-scale, multi-centre RCT would facilitate validation of the efficacy and safety of nebulised ketamine in diverse patient populations and various clinical settings, exploring the use of nebulised ketamine in specific patient populations, such as those with chronic pain or those with comorbid conditions that complicate pain management.

Future research should also focus on refining dosing protocols. While the review suggests that lower doses may be as effective as higher doses in achieving adequate analgesia, more research is needed to establish the optimal dose that balances efficacy with safety.

Further research is needed to compare nebulised ketamine with other commonly used ED analgesics, such as methoxyflurane (Penthrox). Widely used for rapid pain relief in conscious adult trauma patients, Penthrox is effective but associated with notable side effects. Clinical trials report dizziness in over 10% of patients, with other common adverse effects including euphoria, headache, drowsiness, altered taste, cough, nausea, and a sense of intoxication [41]. However, no direct comparative studies between Penthrox and nebulised ketamine have been identified, limiting the ability to directly assess their relative efficacy and safety within the scope of this review.

While the review found no serious adverse events associated with the use of nebulised ketamine, the long-term safety and potential for repeated administration in patients with recurrent pain is unclear. Understanding the long-term safety for patients and those administering nebulised ketamine will be crucial for its widespread introduction into clinical practice.

## Conclusion

In conclusion, this systematic review demonstrates that nebulised ketamine holds significant promise as a non-invasive, effective, and safe analgesic for use in the ED. The data suggests that nebulised ketamine not only effectively reduces pain scores but also does so with a lower incidence of side effects compared to other commonly used analgesics. However, the limited patient numbers, small sample sizes, and methodological inconsistencies across studies underscore the need for more comprehensive research to solidify these findings and overcome the limitations present in the current body of evidence. Further studies are essential to determine the optimal dosing and broader applicability of nebulised ketamine in clinical practice.

## Figures and Tables

**Fig. 8:**
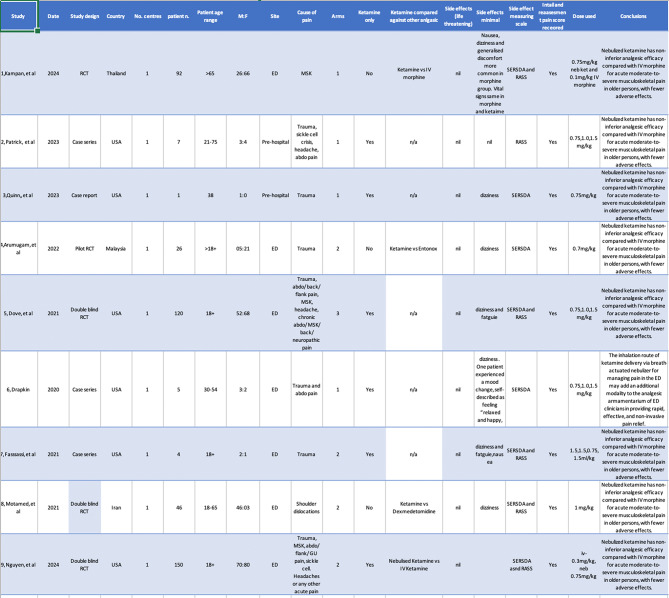
Study characteristics

**Fig. 9:**
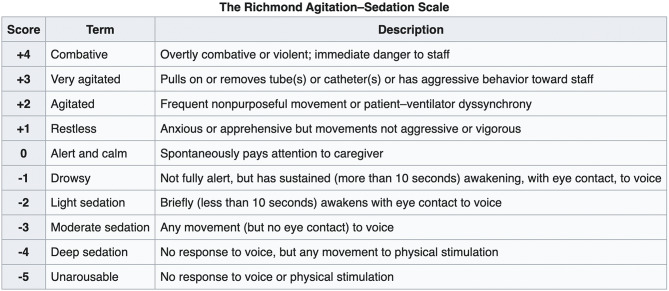
The Richmond agitation-sedation scale

**Fig. 10:**
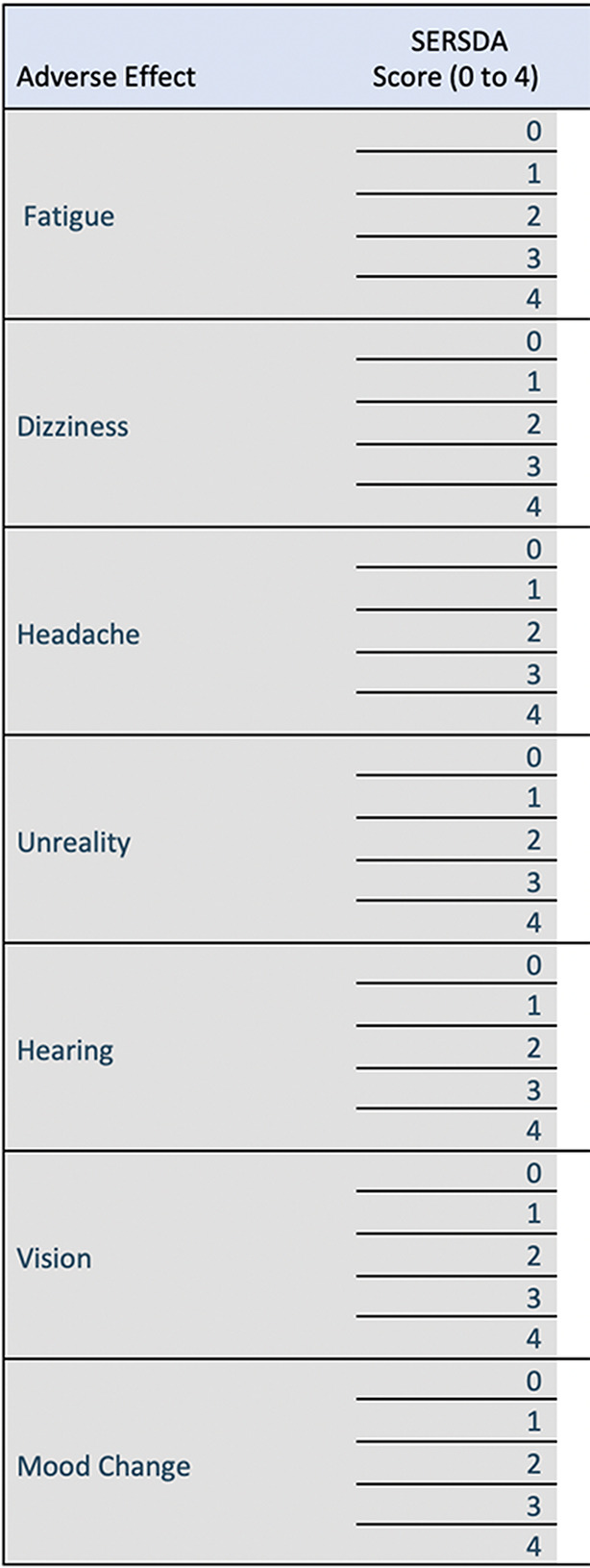
Side Effects Rating Scale for Dissociative Anaesthetics (SERSDA)

## Data Availability

All data generated or analysed during this study are included in the published article.

## Appendix

### Appendix 4:

Raw data

4.1 Outcome 1.

## Abbreviations

BAN-Breath: Actuated nebuliser
BNF-British: National formulary
CASP: Critical appraisal skills programme
ED: Emergency department
GABAA: Gamma aminobutyric acid
IV: Intravenous
NICE: National institute of health and care excellence
NHS: National health service
NMDA: N-methyl-D-aspartate
NRS: Numeric rating score
MeSH: Medical subject headings
PRISMA: Preferred reporting items for systematic reviews and meta-analyses
PROSPERO: Prospective register of systematic reviews
ROB2: Risk of bias version 2
ROBINS-I: Risk of bias in non-randomised studies of interventions
RCT: Randomised controlled trial
RASS: Richmond agitation-sedation scale
SERSDA: Side effects rating scale of dissociative anaesthetics
VAS: Visual analogue scale
WHO: The World Health Organisation
